# DNAcycP: a deep learning tool for DNA cyclizability prediction

**DOI:** 10.1093/nar/gkac162

**Published:** 2022-03-14

**Authors:** Keren Li, Matthew Carroll, Reza Vafabakhsh, Xiaozhong A Wang, Ji-Ping Wang

**Affiliations:** Department of Statistics, Northwestern University, 633 Clark Street, Evanston, IL 60208, USA; NSF-Simons Center for Quantitative Biology, Northwestern University, Evanston, IL 60208, USA; Weinberg College IT Solutions (WITS), Northwestern University, 633 Clark Street, Evanston, IL 60208, USA; Department of Molecular Biosciences, Northwestern University, Evanston, IL 60208, USA; Department of Molecular Biosciences, Northwestern University, Evanston, IL 60208, USA; NSF-Simons Center for Quantitative Biology, Northwestern University, Evanston, IL 60208, USA; Department of Statistics, Northwestern University, 633 Clark Street, Evanston, IL 60208, USA; NSF-Simons Center for Quantitative Biology, Northwestern University, Evanston, IL 60208, USA

## Abstract

DNA mechanical properties play a critical role in every aspect of DNA-dependent biological processes. Recently a high throughput assay named loop-seq has been developed to quantify the intrinsic bendability of a massive number of DNA fragments simultaneously. Using the loop-seq data, we develop a software tool, *DNAcycP*, based on a deep-learning approach for intrinsic DNA cyclizability prediction. We demonstrate DNAcycP predicts intrinsic DNA cyclizability with high fidelity compared to the experimental data. Using an independent dataset from in *vitro* selection for enrichment of loopable sequences, we further verified the predicted cyclizability score, termed *C-*score, can well distinguish DNA fragments with different loopability. We applied DNAcycP to multiple species and compared the *C*-scores with available high-resolution chemical nucleosome maps. Our analyses showed that both yeast and mouse genomes share a conserved feature of high DNA bendability spanning nucleosome dyads. Additionally, we extended our analysis to transcription factor binding sites and surprisingly found that the cyclizability is substantially elevated at CTCF binding sites in the mouse genome. We further demonstrate this distinct mechanical property is conserved across mammalian species and is inherent to CTCF binding DNA motif.

## INTRODUCTION

DNA bendability is a fundamental mechanical property that affects genomic packing and transcriptional regulation across species (1,2). In the context of genome packaging, a 147-bp stretch of eukaryotic DNA is tightly bent around histone octamer to form the nucleosome as the basic unit of chromatin (3,4). By contrast, the entire viral genome undergoes sharp bending to fit into a capsid (2). In the process of transcription, many protein factors bend DNA sequence to form DNA-protein complexes regulating gene expression (2,5–9). For example, it is well known that human TATA-binding protein (TBP) binds to a bent DNA element in the promoter (10,11). Moreover, it is a common feature in both prokaryotes and eukaryotes that DNA bending enables the formation of a small DNA loop that allows juxtaposed transcription factors to regulate transcription (2,12). Beyond its role in biological functions, DNA flexibility is also a key design parameter for DNA-based nanotechnology application such as designing DNA origami, force sensors and other environmental sensors and creating nanomachines (13–16). Thus, to better understand the relationship between intrinsic mechanical properties of double-stranded DNA and its biological applications, it is highly desirable to have a tool for direct quantification of DNA bendability.

Increasing evidence has shown that DNA sequence is a major determinant of DNA flexibility along its central axis due to variations in stacking between adjacent base pairs (17–26). At present, theoretical modeling is not suitable to provide sequence-dependent quantification of intrinsic DNA bendability on a genomic scale (1,27–31). One important practical parameter to quantify DNA bendability, relevant to its biological functions is dsDNA looping efficiency or cyclization efficiency (32). Following the initial cyclization assay, a number of studies involving DNA ligation or single-molecule Fluorescence Resonance Energy Transfer (smFRET) have identified DNA sequences with relatively large bendability or compared relative bendability between DNA of varying lengths and sequences (25,33–35). However, most of them were based on small-scale study or do not provide a direct metric for bendability of every sequence (26,32,33,36,37). In 2020, Basu and his coworkers developed a high-throughput sequencing method to measure the bendability of 90 000 unique DNA sequences in a single experiment (38). In this method named ‘loop-seq’, a library of dsRNA is immobilized on streptavidin-coated beads. After capturing DNA looping through base-pairing between complementary overhangs, RecBCD exonucleases digest unlooped DNA containing free ends, thereby allowing the enrichment of the looped DNA fragments. Both enriched and non-enriched control libraries are subjected to deep sequencing. The cyclizability score is defined as the log ratio of proportion of each sequence species in the loop-seq library that survive digestion of un-looped sequences over the proportion of the corresponding sequence species in the control library without undergoing the same digestion. The intrinsic cyclizability score is subsequently defined by accounting for the tether location effect based on an oscillation model for the raw cyclizability scores.

The loop-seq method for the first time offers a quantifiable measurement of DNA bendability of a vast number of unique DNA fragments. Moreover, the intrinsic cyclizability score it defined provides a very useful metric for DNA bendability that sheds insights into how DNA mechanics underlies various biological observations. Nevertheless, loop-seq experiments are a significant undertaking and costly, particularly when applying this approach to a large mammalian genome. In addition, loop-seq score may be affected by experimental conditions and variables in library design. The cyclizability score from different libraries can only be compared up to an additive library-specific constant even under the same experimental protocol. In practice it would be highly desirable to have a tool that can predict the intrinsic cyclizability accurately and efficiently for any given sequence. In this study, building upon loop-seq experimental datasets (38), we develop a software tool *DNAcycP*, and demonstrate its effectiveness in accurate prediction of intrinsic DNA bendability across multiple species.

## MATERIALS AND METHODS

### Experimental data

We considered five loop-seq data sets from (38) for model training and comparisons, including: (i) *Saccharomyces cerevisiae* nucleosome library of 19 907 different sequences of 50 bp selected from immediate upstream or downstream of the dyads (dyads not included) of 10 000 nucleosomes with highest NCP scores from (39) in *S. cerevisiae* SacCer2 genome; (ii) random sequence library of 12 472 sequences generated with equal expected frequency of A/C/GT; (iii) a tiling library of 82 368 sequences from 576 genes that were selected from yeast genome whose ORFs ends were both mapped with high confidence, among which the first 297 were randomly chosen and the subsequent 279 had highest expression values. For each gene, the +1 nucleosome dyad position ±2000 bp region was first selected, and 50 bp sequences within this region were extracted with tiling spacing of 7 bp; and (iv) yeast ChrV library of 82 404 50 bp sequences tiled with 7 bp spacing.

The fifth data set was from an independent in vitro study for DNA propensity for looping (25). The initial library L0 contained ∼2.4E15 species of 90 bp DNA fragments synthesized randomly. The subsequent libraries L1, L2, …, L6 contained 90 bp DNA fragments that successfully formed loops in previous rounds under different experimental conditions. For L0–L3, the reaction volume remained constant as 2.18 l, while the ligation time monotonically decreased from 30 min, 15 min, 10 min to 4 min sequentially. For L4, L5 the reaction volume was reduced to 500 ml and the ligation time decreased to 1 min and 10 s respectively. Due to the uncertainty caused by a drastic change of ligation conditions in L4 and L5, we only focused our analyses on libraries L0–L3 in the paper. We randomly selected 100,000 fragments from each library to evaluate the prediction of DNAcycP.

### DNAcycP software tool

In this paper we develop a computational tool named *DNAcycP* based on a deep learning model. Recently deep learning methods have achieved remarkable successes in various applications in genomics (40). In particular, the deep convolutional neural networks (DCNN) and deep recurrent neural networks (DRNN) have been widely applied in problems involving DNA sequences such as transcription factor binding sites prediction (41–51) and etc.

**Figure 1.**
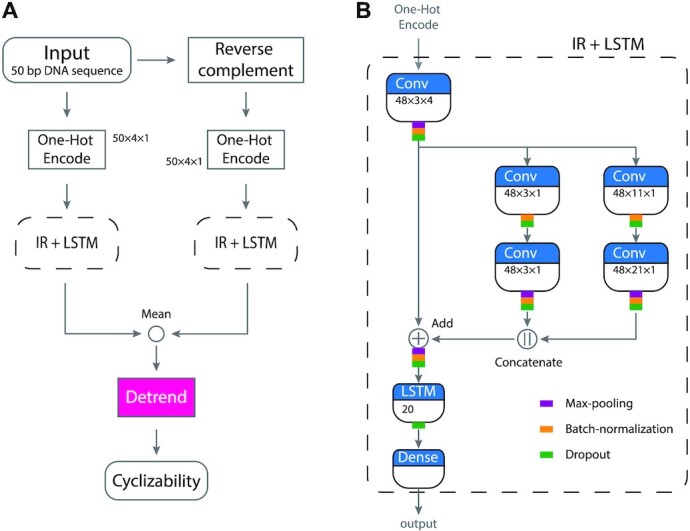
DNAcycP workflow and deep learning architecture. (**A**) Every sequence of 50 bp and its reverse complement are one-hot encoded into a 50 × 4 × 1 tensor, which are subsequently processed independently by the IR + LSTM deep learning architecture. The IR + LSTM outputs of each sequence and its reverse complement are averaged and linearly detrended to output the predicted cyclizability score (*C*-score). (**B**) IR + LSTM architecture model starts with a 3 × 4 two-dimensional convolution layer for dimension reduction of the sequence space from 2D to 1D. The output is fed into an inception module that consists of two branches, each with two serial convolutional layers. One has same kernel size of 3 × 1 and the other has kernels of size 11 × 1 and 21 × 1 respectively. The outputs from the two branches are concatenated. The inception module output is added elementwise back on top of the input of the inception module to form a residual network (or short circuit). Each convolutional layer is equipped with ReLU activation function, followed by max-pooling (except first convolutional layer on each branch), batch-normalization, and 0.2 dropout layers. After a max-pooling, a batch-normalization, and a dropout layers, the output from the residual module is passed onto an LSTM layer with 20 hidden memory units, followed by a dropout. A dense layer follows the LSTM layer with linear activation function for prediction of intrinsic cyclizability score.

The developed DNAcycP takes the one-hot encoding of every 50 bp DNA sequence and its reverse complement as input. The core of DNAcycP is a deep learning architecture pipeline that processes the sequence and its reverse complement separately, the results from which are averaged and detrended to reach the predicted intrinsic cyclizability score (Figure 1A).

Inspired by the work in (52,53) we experimented various deep learning architectures and arrived at an effective model mixed with an Inception-ResNet structure and an LSTM layer (to be called IR + LSTM, Figure 1B). The IR + LSTM architecture starts with a convolutional layer for dimension reduction such that the encoded sequence space is reduced from 2D to 1D. The output is fed into an inception module that contains two parallel branches, each having two sequentially connected convolutional layers with branch-specific kernels to capture sequence features of different scale. The first branch has kernel dimension 3 × 1 for both layers and the second has kernel dimension 11 × 1 and 21 × 1 sequentially. For each output of the convolution layers, the ReLU function is applied, followed subsequently by 2 × 1 max-pooling (except first convolutional layer on each branch), batch-normalization and a dropout with a ratio of 0.2. The stride is equal to 1 throughout. The output of the inception module is combined by concatenation and added back to the input of the inception module to form a short circuit or residual network. After a 2 × 1 max-pooling, a batch-normalization and a dropout layer, the resulting layer is then passed onto an LSTM layer with 20 hidden memory units, followed by a dropout layer with a ratio of 0.2. Finally, the IR + LSTM concludes with a dense layer to predict output with linear activation function. The different kernel dimensions in the inception branches were chosen for the consideration to capture sequence properties including codon, poly-A/T tracks or 10-bp periodicity of dinucleotide motifs that have been shown in the literature to affect DNA sequence flexibility (see Results). The LSTM layer further provides a holistic capturing of sequencing information in the scale of entire input sequence length such as the strength of periodicity of key dinucleotide motifs and their phase angles etc.

We implemented the IR + LSTM pipeline using Keras (https://github.com/fchollet/keras) environment with TensorFlow (54) as backend. The model was trained by stochastic gradient descent algorithm using the rmsprop optimizer with MSE loss function. To prevent over-fitting issue, an early stopping technique, check-point, was used in the 10-fold cross validation, and multiple drop-out layers were deployed in the IR + LSTM architecture. We found a common mean-drift and variance-shrinkage issue with all models considered in this paper (55). The predicted values from different runs often achieved similar correlation with the ground truth but with pronounced overall mean shift and variance shrinkage (see Note 1 in Supplementary Information). To correct this bias, we added a detrend step to remove the linear trend in the finalized model.

## RESULTS

### DNAcycP training and model comparison

To implement IR + LSTM structure in DNAcycP for prediction of intrinsic DNA cyclizability we first determined which loop-seq library to use for model training purpose among: (i) nucleosome library, (ii) random library, (iii) tiling library and (iv) ChrV library as listed above. For a given library the model training was performed under a 10-fold cross-validation framework, i.e. the entire training set of sequences was divided into 10-fold, 9-fold used for model training and the last fold for model testing. Each model resulting from the 10-fold cross-validation was further applied to predict the intrinsic cyclizability score of the other three test libraries. This allowed us to evaluate the model overall performance and its robustness. As the cyclizability scores are only comparable up to an additive library-specific constant, Pearson correlation was used to evaluate the prediction accuracy for external test libraries. Since the four libraries were constructed for different targeted sequences, they may represent different sequence features in the sequence space. We aim to choose one that is most representative that can lead to robust overall performance.

Among four training libraries, IR + LSTM trained based on tiling library achieved the highest prediction accuracy measured by Pearson correlation. For the training data itself, the average Pearson correlation on the testing folds between predicted intrinsic cyclizability score and measured intrinsic cyclizability score from loop-seq score is as high as 0.916. Most remarkably, the prediction accuracy for the three testing libraries, i.e. nucleosome, ChrV and random libraries is 0.893, 0.773, 0.930 respectively, superior to 0.860, 0.767, 0.885, the prediction accuracy achieved on the test folds when models were trained based on the three individual libraries (Supplementary Figure S1a). In addition, the IR + LSTM model trained on tiling library performs much more stable compared to the models trained on other libraries as reflected in the much smaller variance of prediction accuracy observed in the cross-validation. This implicates that the tiling library provides a more robust representation in the sequence space such that the sequence features can be sufficiently learned with the current deep learning architecture.

We also compared IR + LSTM with various architectures from the literature. Some of the results are presented in Supplementary Figure S1b–d (Supplementary Information). The finalized DNAcycP software tool was based on the best-performed IR + LSTM model from the 10-fold cross validation trained on the tiling library. It is worth noting that the training data (tiling library) was standardized with 0 mean and unit variance. The standardization does not change the prediction accuracy in terms of correlation, but it provides two advantages. To facilitate the discussion in the following context, we term the predicted intrinsic cyclizability ‘*C*-score’ and the experimentally determined intrinsic cyclizability score ‘loop-seq score’. Firstly, the *C*-score directly indicates the statistical significance relative to the population mean in yeast genome, i.e. a *C*-score of 1.96 in yeast genome tells it is 1.96 standard deviation above genome average or it corresponds to ∼97.5% quantile of the genome-wide *C*-score distribution. Secondly as the loop-seq scores from different experiments are only comparable up to an additive library-specific constant, the zero-mean in the training data provides a convenient unified baseline (i.e. the mean of yeast genome intrinsic cyclizability score defined as 0) that can be used to gauge the *C*-score predicted for other species or other sequence libraries.

### DNAcycP predicts intrinsic DNA cyclizability with high fidelity

**Figure 2.**
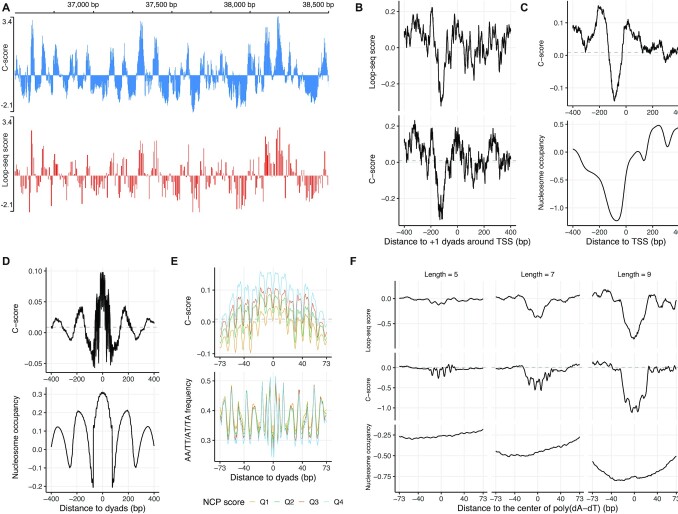
DNAcycP accurately predicts loop-seq quantified intrinsic cyclizability score on yeast genome. In all figures hereafter throughout this manuscript the NCP scores and weighted occupancies are standardized over the genome with mean zero and unit variane for convenience of comparison with the genome average. For all figures with *C*-score plot, the horizontal dash lines (grey) represent the genome average *C*-score for the species under consideration. (**A**) Predicted intrinsic cyclizability score (*C*-score) versus loop-seq quantified intrinsic cyclizability score (loop-seq score) in an example region of yeast ChrV shows high consistency with Pearson correlation = 0.908 (0.773 in entire ChrV). As DNAcycP was trained based on standardized intrinsic cyclizability score, for convenience of comparison, the loop-seq score from ChrV was also standardized to have mean 0 and std = 1. (**B**) Mean loop-seq score and *C*-score aligned at +1 nucleosome dyads downstream of TSSs from yeast ChrV. The unique nucleosome map was from (39). (**C**) Mean *C*-score and nucleosome occupancy around 3017 TSSs from all chromosomes. (**D**) Mean *C*-score and nucleosome occupancy aligned at unique nucleosome dyads from all chromosomes. (**E**) Mean *C*-score and dinucleotide AA/AT/TA/TT frequency aligned at nucleosome dyads. The four curves correspond to four quartiles of the unique nucleosomes based on their NCP scores. (**F**) Average loop-seq scores (upper), *C*-scores (middle) and nucleosome occupancy (lower) around poly(dA:dT) tracts on ChrV for tract length exactly = 5, 7, 9 bp.

We first investigated whether the predicted intrinsic cyclizability score, *C*-score, can faithfully reproduce results from the experimentally measured intrinsic cyclizability score by loop-seq data in (38). To compare *C*-score and loop-seq score, we first visualized the ChrV data on yeast genome browser. Figure 2A exemplifies a typical region that shows high consistency between *C*-score and loop-seq score (Pearson correlation = 0.908 in this region and 0.773 on entire ChrV. Note loop-seq score is at resolution of 7 bp and *C*-score is at 1 bp). This high consistency was further illustrated in the average *C*-score and loop-seq score patterns around + 1 nucleosome dyad at 125 TSSs from ChrV (Figure 2B). We then expanded this analysis to entire genome by selecting 3017 genes that have well annotated TSS sites (39) and plotted the average *C*-scores around the TSSs. The *C*-scores displayed a substantial dip upstream of TSS (Figure 2C), confirming the low bendability of DNA sequence in nucleosome depleted region concluded in (38) based on loop-seq data of 576 selected genes.

We next examined how *C*-score correlates with genome-wide nucleosome positioning. When aligned at the dyads of 64 038 unique nucleosomes from (39), the *C*-score displayed a synchronized phasing pattern as the nucleosomes occupancy (Figure 2D). We further divided the nucleosomes into four quartiles according to their nucleosome center positioning (NCP) score and calculated the average *C*-score of each quartile, as well as the corresponding AA/TT/TA/AT dinucleotide frequency (Figure 2E). The *C*-score in the nucleosome region shows a positive correlation with the magnitude of nucleosome center positioning (NCP) score. The base pair resolution of *C*-score enables to reveal more details of the cyclizability score that were not revealed in the loop-seq data due to its limitation of resolution. We observe a pronounced fine 10-bp periodic pattern of *C*-score where the *C*-score peaks align with the periodic AA/TT/TA/AT dinucleotide motif peaks within nucleosome region (Figure 2E). In contrast to these dinucleotide sequence motifs, the poly(dA:dT) tracts are well known to deplete nucleosome occupancy for being too stiff (56,57). We plotted the average *C*-scores and loop-seq scores around poly(dA:dT) tracts on ChrV for tract length exactly = 5, 7, 9 bp. Similar to the loop-seq data, the *C*-score revealed a radical decline of intrinsic cyclizability as the tract length increased from 5 to 9 bp (Figure 2F), so was the nucleosome occupancy. This result demonstrates that the DNAcycP model has successfully identified such sequence features that significantly impact DNA cyclizability. Taken together we conclude the DNAcycP can reproduce the intrinsic cyclizability of DNA fragments from the loop-seq assay faithfully.

### Validation of *C*-score with independent experimental dataset

**Figure 3.**
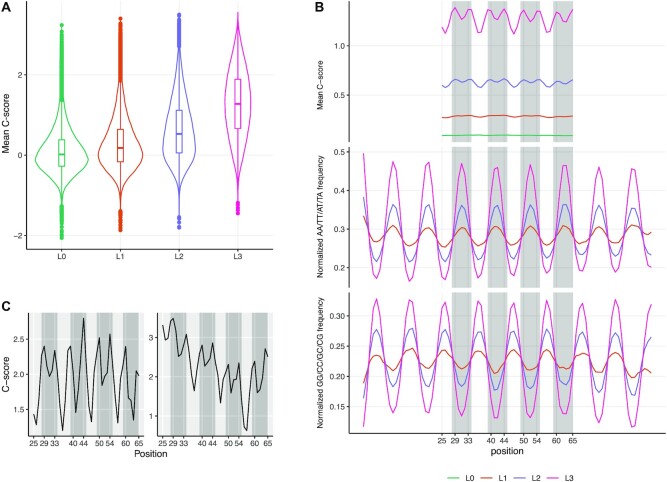
Predicted intrinsic cyclizability score on an in vitro selected loopable DNA sequence dataset (25). From each library L0–L3, 100 000 sequences were randomly selected. For each sequence of length 90 bp, *C*-score was predicted for every subsequence of 50bp using a sliding window. The score at 25 or 65 represents the 50-bp DNA fragments starting from position 1 or 41 respectively. (**A**) Violin- and box- plots of the mean *C*-score in 90 bp sequences in L0 to L3. (**B**) Mean *C*-score and the average AA/AT/TA/TT and GG/GC/CG/CC frequency along all selected sequences in each library. (**C**) *C*-score track plots for two randomly selected sequences from L3.

To validate *C*-score as a meaningful quantification of intrinsic DNA bendability independently from the loop-seq data, we applied DNAcycP to the experimental dataset (dataset 5 above) using a different cyclization assay (25). In this system, highly cyclizable DNA sequences were selected and enriched from a very large starting library of random DNA sequences (90 bp), under an experimental condition strongly favoring intramolecular DNA ligation. Because of extraordinary complexity of the libraries (25), this selection scheme did not actually offer a quantifiable measurement of intrinsic bendability for individual DNA sequences but provided libraries of DNA sequences having varying degrees of enrichment for DNA sequences with higher loopability. Thus, we ask whether the *C*-score can distinguish the DNA fragments in these libraries that were progressively selected for looping propensity. For each sequence of 90 bp, we computed the *C*-score for every 50 bp using a sliding window of step size 1 bp. The resulting average *C*-scores of the 50-mers centered at positions from 25–65 (note: score at position 25 is for DNA fragment from position 1 to 50, etc.) shows a substantial progressive increase from library L0 to L3, with median *C*-scores increasing from –0.017 to 1.277 (Figure 3A), confirming the increasing bendability of 90-mer DNA fragments. Interestingly we found four pronounced bi-modal peaks in L2 and L3 besides the elevated baseline compared to L0, evenly spaced by ∼10.5 bp (Figure 3B). Rosanio et al (25) found that the increasing looping propensity in L0–L3 is positively correlated with AA and GG dinucleotide periodicity. We reproduced this plot by including all A/T or G/C dinucleotide (i.e. AA/TT/TA/AT, GC/CG/GG/CC) and found that the four pronounced *C*-score bi-modal peaks in L2 and L3, centered at positions ∼31, 42, 52, 63 respectively aligning with the AA/TT/AT/TA motif positions (and anti-phase with GC/CG/GG/CC, Figure 3B). This observation echoes the result from yeast data from Figure 2E, and supports the ‘wedge’ model that periodic positioning of such dinucleotide motifs contributes significantly to inherent curvature of DNA (58); and the rotational phase angle of the motifs in the sequence further affects the cyclizability in a finer scale. We plotted the *C*-score track of randomly selected individual sequences from L3 and two of these sequences are shown as examples in Figure 3C. While both sequences displaying high average cyclizability, the peak locations of *C*-scores distribute within the entire interval and vary between sequences. The observed broad distribution of intrinsic DNA bendability is consistent with a recent Cryo EM study showing that closed circular 94 bp DNA minicircles are smoothly bent without sharp kinks (59). Therefore, our verification analysis further demonstrates that the *C*-score provides a direct metric to measure intrinsic bendability of DNA sequence on a relative and quantitative scale.

### DNA cyclizability varies between species

**Figure 4.**
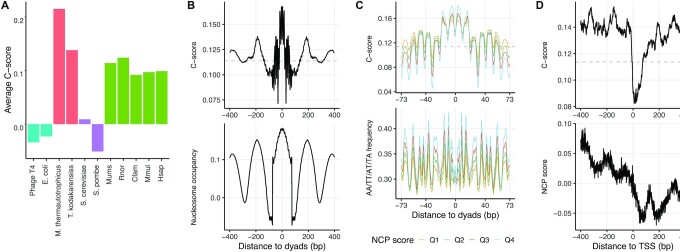
DNA intrinsic cyclizability across species. (**A**) Bar charts of average *C*-score of selected species. For non-mammalian species the *C*-score was averaged over the entire genome, and for the five mammalians *C*-score was calculated based on Chromosome 1 for each species. (**B**) Mean C-score and nucleosome occupancy aligned at unique nucleosome dyads from all chromosomes of mouse genome from (60). (**C**) Mean *C*-score and dinucleotide AA/AT/TA/TT frequency aligned at nucleosome dyads of mouse genome. The four curves correspond to four quartiles of the unique nucleosomes based on NCP scores. (**D**) Plots of *C*-score and nucleosome occupancy averaged over 20 811 TSSs of mouse genome.

Unlike the loop-seq assay where the measured intrinsic cyclizability score may be affected by experimental protocol or library complexity, the *C*-score predicted by DNAcycP provides a unified baseline that allows direct comparison of cyclizability across libraries or species. We applied DNAcycP to multiple species ranging from bacteria, archaea, to eucarya, including *Escherichia coli*, *Escherichia virus T4* (bacteriophage), *Methanothermobacter thermautotrophicus*, *Thermococcus kodakarensiss*, *S. cerevisiae*, *Schizosaccharomyces pombe*, *Canis lupus familiaris* (dog), *Macaca mulatta* (monkey), *Rattus norvegicus* (rat), *Mus musculus* (mouse) and *Homo sapiens* (human). Interestingly, the genome-wide mean *C*-scores from all selected mammal species are higher than the two bacteria (*E. coli* and *bacteriophage*) and the two fungi species (*S. cerevisiae* and *S. pombe*) (Figure 4A, Supplementary Table S1). Such differences are statistically significant due to extremely large sample size. For example, the *P*-value for *S. cerevisiae* and dog comparison is < 2.2E–16 (other *P*-values are not listed). Worth noting that the two archaea species display the highest *C*-scores among the species we have analyzed.

Using the predicted cyclizability we investigated whether the observed relationship between DNA cyclizability and nucleosome positioning in yeast is preserved in other species. Because the high-resolution positioning map of nucleosomes is essential for comparison, we focused on the mouse genome using the unique nucleosome map from (60). Indeed we found the *C*-score around the dyads of unique nucleosomes has a synchronous phasing pattern of nucleosome occupancy (Figure 4B), and *C*-score is positively correlated with the AA/TT/TA/AT dinucleotide frequency (Figure 4C). Like in yeast, four major *C*-score peaks were observed, roughly ∼ 3/4, 15 bp away from both sides of the dyad, suggesting a ∼80 bp region of relatively high cyclizability DNA sequence around nucleosome dyad (note: *C*-score at position 15 from dyad measures the cyclizability of fragment from position -9 to 40). A recent study showed that strong sequence affinity is necessary for wrapping ∼80 bp DNA around (H3–H4)_2_ tetramer in initial stage of nucleosome forming (61). The observation from yeast and mouse genomes concurs that cyclizability may present one essential aspect for DNA affinity for histone binding to form the initial tetrasome, where the positioning of periodic dinucleotide motifs could be a main contributing sequence feature. In contrast with the yeast result (Figure 2C), no significant reduction of *C*-scores is observed upstream of TSS, consistent with our previous finding that fragile nucleosomes occupy the previously designated nucleosome depleted region (Figure 4D). Instead, the NCP scores exhibit a big dip of nucleosome occupancy at about 50 bp downstream of TSS, accompanied by a sharper decline of DNA cyclizability in the immediate downstream of TSS. The combined results from yeast and mouse strongly support that the intrinsic DNA mechanics plays an essential role in determination of the global nucleosome positioning in eukaryotic species.

### DNA cyclizability at transcription factor (TF) binding sites

**Figure 5.**
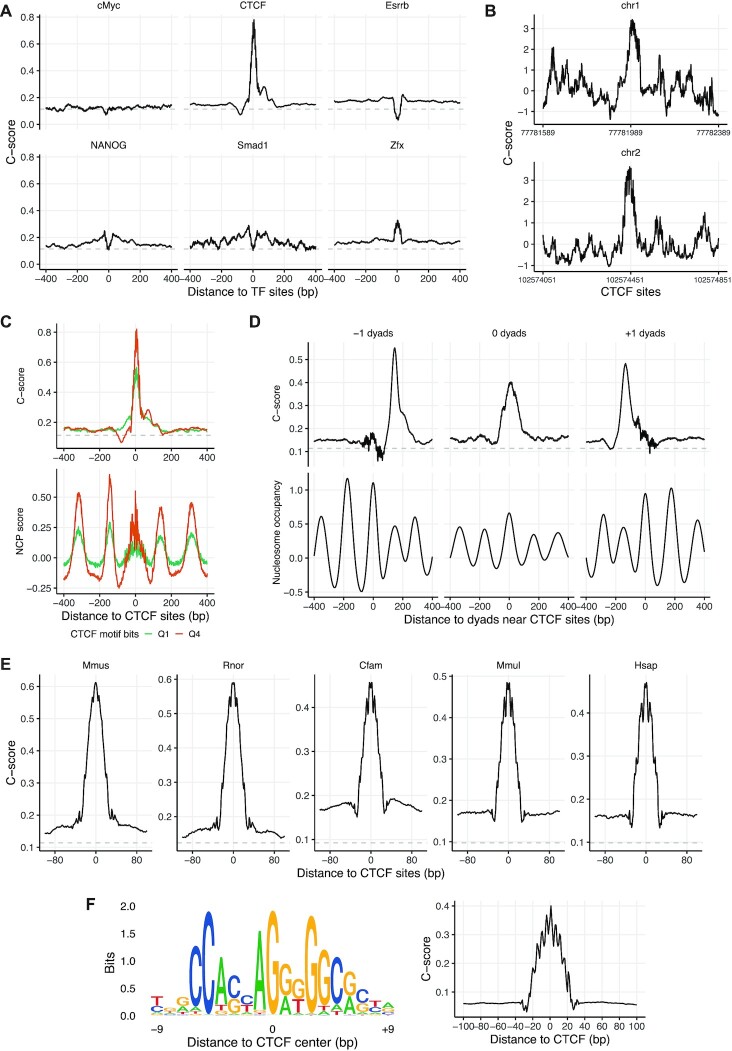
Predicted cyclizability aligned near transcription factor binding sites. (**A**) Mean *C*-score around selected transcription factor binding sites on mouse genome. The TF binding sites of mouse were from ChIP-Seq data (GSE11431, (62)). The exact TF binding sites were refined by scanning the ChIP-seq peak vicinity region using motif model of the TFs (same for (E) below). (**B**) Two selected CTCF binding sites at ChrI:77 781 989 and ChrII:102 574 451 show two pronounced *C*-score peaks. (**C**) *C*-score (upper panel) and NCP-score (lower panel) averaged at the CTCF binding sites. Plotted are for the lowest and highest quartiles (Q1 and Q4) based on CTCF binding site motif score. (**D**) Mean *C*-score and nucleosome occupancy at the −1, 0 and + 1 dyads of the nucleosomes around CTCF sites on mouse genome (unique nucleosome maps from (60)). The ‘0’ nucleosome dyad is defined as the center of the nucleosome in the unique map that covers the given CTCF binding site, i.e. nucleosome center resides within ±73 bp of CTCF binding site. The ‘−1’ and ‘+1’ nucleosome dyads refer to nucleosomes that do not cover a CTCF binding site, but their centers are within 221 bp upstream and downstream respectively. (**E**) Mean *C*-score around CTCF binding sites mapped from liver tissue on five difference species: human (Hsap), dog (Cfam), monkey (Mmul), mouse (Mmus), rat (Rnor) from a separate study (E-MTAB-437, (63)). (**F**) The 19-bp CTCF motif logo plot (left panel) and the mean *C*-score over simulated CTCF motif sequence + extended random sequences (right panel). Using the motif model, we first simulated 19-bp CTCF motif sequence and then extended it to both sides by a random model with equal length until reaching 201 bp. The mean *C*-score was averaged over 100 000 simulated sequences.

In addition to its role in nucleosome formation, DNA cyclizability can directly affect the binding of TFs to their targets. It is well known that a number of TFs preferentially bind to DNA in a bent conformation (2). For many TFs, it remains an open question as to how DNA bending may influence the formation of TF–DNA complexes. We used the genome-wide *C*-score map to investigate how DNA cyclizability may contribute for transcription factor (TF) binding. Taking advantage of available ChIP-Seq datasets for TFs from mouse embryonic stem cells (62), we compared the landscape of accumulative *C*-score around 13 TFs. These include cMyc, CTCF, E2f1, Esrrb, NANOG, Smad1, Zfx, Oct4, Sox2, Stat3, nMyc, Klf4 and E2f1 (Figure 5A and Supplementary Figure S2a). For most TFs, the *C*-scores show statistically significant but only relatively mild changes in the ±200 bp region or at the exact TF binding sites compared to the genome average. However, we surprisingly found that at the exact factor binding sites for CTCF, the cyclizability is substantially higher. When we zoomed in to individual sites, the *C*-scores at some CTCF sites are dramatically elevated as shown in two examples Figure 5B. Therefore, it appears that CTCF strongly favors binding to a bendable target DNA.

We further investigated this unexpected mechanical feature of CTCF binding sites from two aspects. First, we asked whether nucleosome positioning around CTCF sites contributed to higher DNA cyclizability. We have previously showed that CTCF and nucleosomes co-occupy the same DNA target sequences (60). We sorted CTCF sites into four quartiles based on CTCF motif score and compared the *C*-score and NCP score between groups (Figure 5C, Supplementary Figure S2b). At these sites, higher *C*-score correlates with higher CTCF motif score, so is with higher NCP score (Figure 5C) (see latter also in Figure 4B). To disentangle whether the observed high *C*-score is due to nucleosome sequence features or CTCF motifs, we first compared the *C*-score at ±1, 0 nucleosome dyads relative to CTCF, where 0 nucleosome is defined as one in the unique map whose dyad is within ±73 bp of CTCF, and ±1 nucleosome as the immediate next nucleosome beyond ±73 bp of CTCF respectively. Clearly the enrichment of *C*-score at 0 dyad (Figure 5D) or exact CTCF site (Figure 5A) is many folds as large as that at −1/+1 dyad, suggesting the sequence features that facilitate CTCF binding are the predominant contributor of *C*-score at CTCF sites.

Next, we tested whether the *C*-score pattern associated with CTCF is a universal feature across species. We compiled the CTCF ChIP-Seq maps from the liver tissue of five mammalian species (63), including human (Hsap), dog (Cfam), monkey (Mmul), mouse (Mmus) and rat (Rnor). Indeed, the substantially enriched *C*-score around CTCF is well conserved in all five species (Figure 5E). Lastly, we asked what sequence features contributes to substantially enlarged *C*-score at CTCF sites using simulation. We first simulated 100 000 19-bp CTCF motif sequences using the motif model from https://jaspar.genereg.net/matrix/MA0139.1/, and then extended each sequence by adding same-length random sequences to both ends until reaching length of 201 bp. The predicted *C*-score of these simulated sequences shows a pronounced peak at CTCF sites (Figure 5F), but slightly lower than observed in Figure 5A, confirming that most of the enriched *C*-score observed in vivo is attributable to the intrinsic CTCF motif sequence features.

### Simulation study

One pronounced sequence feature associated with high cyclizability revealed by loop-seq and looping propensity data is the presence of 10-bp periodic dinucleotide motifs. It remains unclear to what extent such motifs may account for the enriched DNA cyclizability. Using DNAcycP, we performed two simulations as follows. Firstly, we randomly selected 10 000 sequences from L3 library whose predicted *C*-scores were above the 90th percentile of the set. Based on the alignment of the 10,000 sequences, we trained a first order, a second order and a third order time-dependent Markov Chain models, from which we simulated 10 000 90-bp sequences for each. Such time-dependent Markov chain models are effective to capture the *k*-mer distribution along the sequence for *k* up to the Markov chain order +1. Additionally, we simulated 10 000 90-bp random sequences based on the marginal A/C/G/T composition in the selected sequences. Secondly from the yeast unique nucleosome map, we selected 6404 nucleosomes whose average *C*-score in the dyads ±25 bp positions (which represent the average cyclizability in the region of dyads ±50 bp) was above the 90th percentile of the entire set. Like in the first simulation we simulated four sets of 10 000 147-bp sequences following the first, second, third order time-dependent Markov Chains and random models respectively.

**Figure 6.**
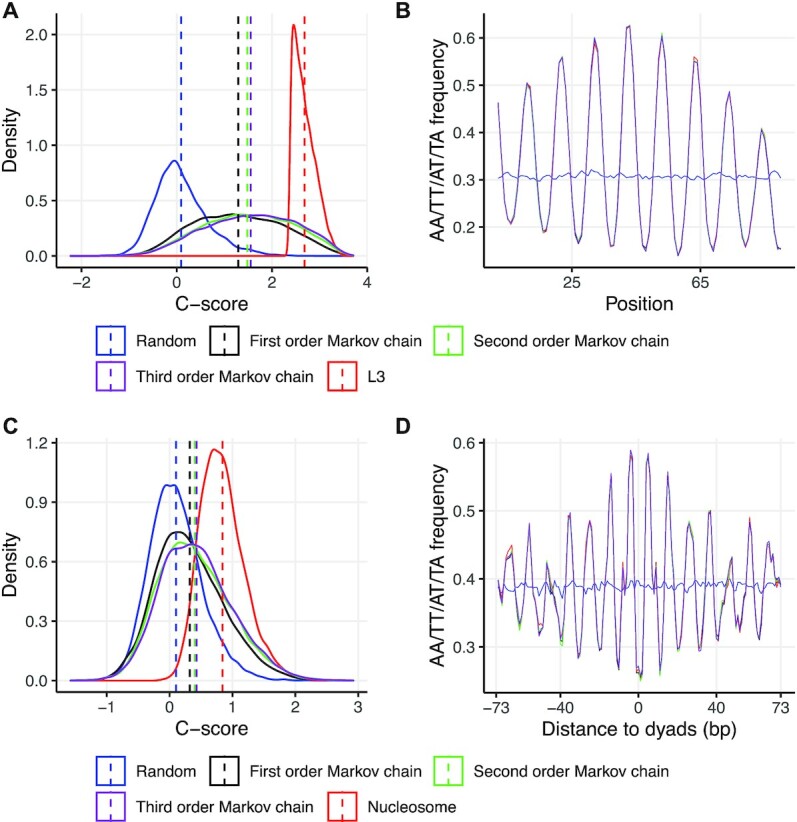
Simulation study. (**A**) *C*-score distributions for simulated sequences based on L3 data. A random sample of 10 000 sequences drawn from L3 library with *C*-scores above the 90th percentile of L3. Plotted are *C*-scores for this selected set (red); 10 000 90-bp sequences simulated from a random model based on the marginal A/C/G/T composition in the selected L3 set (blue); 10 000 90-bp sequences simulated from first/second/third order time-dependent Markov chain models trained from the 10,000 selected L3 set (black/green/purple respectively). (**B**) Average AA/TT/TA/AT dinucleotide frequency from five sets listed in (A). (**C**) *C*-score distributions for simulated sequences based on the nucleosome data. 6,404 nucleosomes were selected from the unique map of yeast whose average *C*-scores in dyad ±25 bp region were above the 90th percentile of the entire set. Sequences were simulated in the exact same way as in L3 data above except the sequence length was 147 bp. (**D**) Average AA/TT/TA/AT dinucleotide signals from five sets listed in (C).

Unsurprisingly in both simulations the sequences from the Markov Chain models perfectly preserved the average periodic AA/TT/TA/AT dinucleotide signal as in the parental sequence sets (so were other dinucleotides, not presented), while not in the random sequence case (Figure 6A, B). In the L3-derieved data, the average *C*-scores for the sequences from the random, first/second/third order Markov chain models and sampled L3 sequences were 0.09, 1.29, 1.48, 1.55 and 2.68 respectively, suggesting that dinucleotide motifs may contribute up to 46% of the increased average *C*-score above the random model. Interestingly the second or third order model, which accounts for the position-dependent tri-/tetra-nucleotide distribution, further raised 6% or 3% of *C*-score over the first/second order model respectively. Likewise, in the nucleosome-derived data, the mean *C*-score for sequences from the random, first/second/third order Markov chain models and the sampled nucleosome sequences were 0.10, 0.32, 0.40, 0.43 and 0.84 respectively, suggesting the dinucleotide motifs may contribute up to 30% of the enlarged *C*-score compared to the random model (Figure 6C, D). Accounting for tri-/tetra-nucleotide signals further resulted in 11% or 4% increment of *C*-score compared to the random model.

These results suggest that such position-specific *k*-mer motifs (up to *k* = 4) may account for ∼50−60% of the enriched cyclizability compared to random models. As the number of parameters in the time-dependent Markov chain increases exponentially with the order, good training of it demands large number of sequences. Furthermore, the increment of average *C*-score appears to attenuate rapidly as Markov chain order reaches 3. Higher orders of models were not pursued in this simulation. Instead, we hypothesize remote inter-positional DNA features that cannot be accounted for by Markov chain models may play an important role to account for the gap of cyclizability score observed from the real and simulated sequences. The high prediction accuracy of DNAcycP demonstrated the capability of deep learning models in learning such features to fill in the prediction accuracy gap beyond *k*-mer models.

### DNAcycP software tool

**Figure 7.**
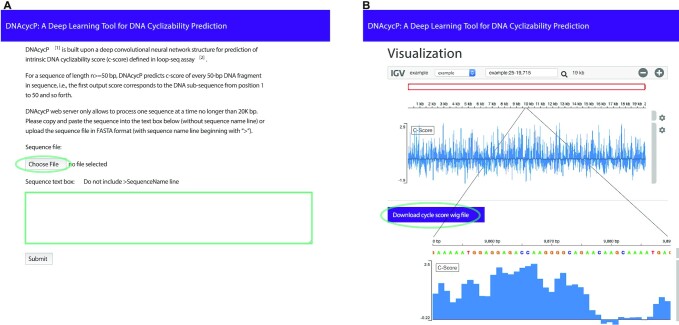
DNAcycP web server. (**A**) A screenshot of DNAcycP web server interface available at http://cyclizability.stats.northwestern.edu. The user can either submit a single sequence up to 20 000 bp in FASTA file or copy and paste the DNA sequence directly into the text box. (**B**) Visualization of DNAcycP predicted *C*-score by embedded IGV genome browser and data downloading. User can zoom in to see local details of the *C*-score distribution or download the *C*-core in WIG format.

Quantitative measurement of DNA mechanical properties such as cyclizability has broad and important applications in biology and DNA nanotechnology (1,16). The recently developed loop-seq method provides the first large-scale practical quantification of DNA cyclizability (38). In this study, we have leveraged these valuable datasets and developed a deep learning approach to predict DNA cyclizability solely based on sequence. To facilitate easy access and usage of DNAcycP, we implemented DNAcycP software tool in two formats, a web server available at http://DNAcycP.stats.northwestern.edu for real-time prediction and visualization of *C*-score up to 20 000 bp (see screenshots of web server in Figure 7), and a standalone Python package available for free download from https://github.com/jipingw.

## DISCUSSION

High throughput loop-seq assay provides the first quantitative measure of DNA cyclizability (38). The landscape of intrinsic cyclizability derived from the experimental data based on yeast genome has greatly improved our understanding of how DNA mechanics contribute to nucleosome organization. In this study, the same genome-scale datasets enable us to develop a deep-learning model that can faithfully reproduce the intrinsic cyclizability quantified by loop-seq experiments. More importantly, although DNAcycP was trained based on yeast tiling library, it achieves desired prediction accuracy and effectiveness for external data sets, particularly for the random library and the looping propensity data where sequences were randomly generated and carry no coding information, indicating that DNAcycP can be applied to other species.

When we applied DNAcycP to predict intrinsic DNA cyclizability for different species, several interesting features have emerged. First, in line with the recent findings from yeast (38), DNA cyclizability correlates with the chemically defined high-resolution nucleosome positioning information in the mouse genome, namely, the central region of nucleosomes favors DNA sequences susceptible for bending to form stable interaction with H3−H4 tetramers. Worth noting that among species we analyzed, the two archaea species exhibit the highest *C*-scores. It is known that *M. thermautotrophicus* and *T. kodakarensiss* have special nucleosome structure that involves only ∼60 bp DNAs to wrap around (H3 + H4)_2_ tetramer (64,65). It is possible that relative larger cyclizability might contribute to sharp bend of DNA required to form special architecture of nucleosomes in archaea species. Second, nucleosome depleted regions in general correlate with lower DNA bendability in the genome (Figure 2F), further supporting the idea that DNA cyclizability is a contributing factor in nucleosome positioning. Third, various TFs may have different preferences for DNA bendability at their binding sites. CTCF binding motif displays an unexpected feature with high DNA bendability that are conserved in mammals (Figure 5). While the biological significance of this surprising feature remains unknown, we speculate that this mechanical property may be related to the role of CTCF in promoting loop extrusion (66). Alternatively, our recent chemical mapping data showed CTCF may act as a pioneer factor that binds to DNA sequence on the surface of a nucleosome (60,67). If so, a sharply bent CTCF motif may have evolved to accommodate the formation of CTCF-nucleosome complex. Future experiments will be necessary to explore these possibilities.

On the experimental side, the current DNAcycP is rooted in loop-seq data from yeast data. As more experimental datasets from different species become available, more trainings and tests will be performed to verify and potentially improve the current version of DNAcycP. One important future direction is to acquire loop-seq data that will allow us to test how epigenetic modifications such as DNA methylation will affect DNA bendability (68,69). Once such data are generated, a new version of methyl-DNAcycP will be implemented to evaluate and predict the effect of DNA methylation on DNA mechanics. Additionally, at the technical level, the current version carries some limitations that loop-seq assay may have. For example, the *C*-score is only defined based on every sequence fragment of 50 bp. DNAcycP cannot be used to predict cyclizability for shorter sequences.

On the computational side, DNAcycP was implemented based on a structure with a hybrid of inception and residual modules and an LSTM layer. Combining convolutional and LSTM layers has been often used in modeling DNA sequences (38,48,52). The LSTM layer is intended to find the relation of most relevant features summarized from the convolutional layers, such as the recurrent or periodic key dinucleotide signals that have been proven predictive of the sequence bendability. The inception module, i.e. the parallel branches, allows us to use different filter sizes to extract important sequence features at different scale, which are subsequently concatenated and passed onto next layer. The residual network structure is often used to effectively recycle the useful information missed by the convolutional layers to prevent decay of performance due to deeper convolution layers. We ended up with IR + LSTM architecture for its competitive performance and relative better computing efficiency compared to many models we experimented (Supplementary information). Due to the vast amount of available architectures or model variants, exhaustive search of models is implausible. One biggest advantage of deep learning model lies in its ability to explore the model space to create a complex and nonlinear model for high prediction accuracy. We observed a mean-drift issue in the proposed and other deep-learning structures implemented in the Keras environment and corrected it by a linear detrending procedure. However, we are unable to directly verify whether this detrending procedure is transferrable to external test data or data from other species because the measured cyclizability score is subject to a library-specific constant. One possibility for future loop-seq experiments is to include a robust set of spike-in controls, based on which the cyclizability scores across libraries can be normalized. Once such data is available further investigation of this matter will be performed and the online DNAcycP tool will be updated. Another aspect of future work is to quantify the uncertainty of the *C*-score prediction. The prediction uncertainty may arise from two aspects in the deep learning models. One from the uncertainty of model training/estimation and the other from random noise. For the model training/estimation side, the complexity of the deep learning model prevents us from quantifying the uncertainty analytically. One could fit the deep learning model many times and apply the resulting models to the data under prediction to obtain uncertainty measure, which though could be computational expensive. For the second aspect, it is questionable whether a constant Gaussian noise can be assigned. Given this, we leave this as an open question for future work.

Unique to this work, we have by serendipity found that CTCF binding motif is an inherently flexible DNA sequence, only representing a tiny portion of sequence space that account for varying degrees of DNA bending capacity. Consistent with a long-standing notion in the field, our DNAcycP prediction and simulation studies have verified that the 10-bp periodic dinucleotide signals is one contributing sequence features for cyclizability of DNA sequence. Our simulation study demonstrated that the position-dependent *k*-mer motifs may account for 50–60% elevated cyclizability compared to the random model. The Markov chain models can well represent the local sequence dependence (i.e. *k*-mer) because of the Markovian property, while they cannot effectively model the dependence in longer ranges such as 20 or 30 bp away given the limitation of orders. The deep learning models excel in this regard through convolution layers with appropriate filter dimension or LSTM layers. The tradeoff is its black box nature and lack of interpretability. Besides this known sequence feature, a combination of DNAcycP and other computational methods is under investigation for deeper understanding of determinants of DNA intrinsic bendability in the future.

## DATA AVAILABILITY

DNAcycP web server available at http://DNAcycP.stats.northwestern.edu for real-time prediction and visualization of *C*-score up to 20K bp, and a standalone Python package available for free download from https://github.com/jipingw. R/Python codes for data processing are available upon request.

## Supplementary Material

gkac162_Supplemental_File
