# Supplementary material for: DNAcycP: a deep learning tool for DNA cyclizability prediction
Source: Nucleic Acids Res. 2022 Mar 14;50(6):3142–54. doi: 10.1093/nar/gkac162 (PMC8989542; doi:10.1093/nar/gkac162)
Supplement: gkac162_Supplemental_File [file gkac162_supplemental_file.docx]

**Supplementary Information**

**DNA Cyclizability Prediction**

Keren Li, Matthew Carroll, Reza Vafabakhsh, Xiaozhong Wang and Ji-Ping Wang

**Supplementary Note 1: Deep learning model training and comparison**

We first considered which of the four libraries to use for model training purpose, including Cerevisiae Nucleosomal (CN) Library (19,907 sequences), Random Library (12,472 sequences), Tiling Library (82,367 sequences), and ChrV Library (82,404 sequences). As the intrinsic cyclizability score from different libraries are only comparable up to a library specific constant and these libraries may represent different sequence features in sequence space, we cannot directly merge them for model training purpose. Thus we trained the IR+LSTM model based on each individual library and examined the performance of the trained model on the testing data, i.e., testing folds in 10-fold cross-validation in the training library and the three other testing libraries.

Among four training libraries, IR+LSTM trained on tiling library outperformed the models trained on other libraries in accuracy in terms of Pearson correlation. For the training data itself, the average Pearson correlation on the testing folds between predicted C-score and measure loop-seq score is as high as 0.916 (Supplementary Fig. 1a). Most remarkably, the prediction accuracy for the three testing libraries, i.e., CN, ChrV and Random Libraries is 0.893, 0.773, 0.930 respectively, superior to 0.860, 0.767, 0.885, the prediction accuracy achieved on the validation folds when models were trained based on these three libraries respectively. In addition, the IR+LSTM model trained on tiling library achieves better stability (i.e., small variance) in prediction accuracy compared to models trained on other libraries.

For comparison purpose, we benchmarked IR+LSTM model with four different neural network architectures adapted from (1) including: a deep convolutional model of three ResNet structures in sequence including nine CNN layers (DCNN), a convolutional model with three different dilated CNNs to capture long range dependencies (Dilated CNN), a convolutional model with three CNN layers and a time distributed dense layer (Time Distributed CNN), and a mixed convolutional neural network by adding an LSTM layer to the end of three CNNs (CNN+LSTM). The corresponding scripts of all models discussed in this paper are available to download from <https://github.com/jipingw>.

Similar as in IR+LSTM model, all the four deep-learning structures trained on the Tiling Library had significantly better performance than models trained other libraries (results not shown). Thus, we focus on comparing IR+LSTM with the four benchmark structures trained based on the Tiling Library. Among the four benchmark structures, the DCNN model also performs competitively relative to other three (Supplementary Fig. 1b). Compared to DCNN, IR+LSM model achieved slightly better average prediction accuracy in all four libraries, but much better stability in terms of variance of prediction accuracy for the test folds in training data, as well as in the external test libraries. This was further reflected in the MSE metric, where IR+LSTM model resulted in much smaller MSE in the testing folds in the 10-fold cross-validation than DCNN (Supplementary Fig. 1c). Note that MSE is not evaluated for prediction of external testing libraries (CN, Random and ChrV), as the intrinsic cyclizability score between libraries can only be compared up to a constant difference.

The relatively larger MSE in DCNN and other models is partially due to a mean-shift phenomenon, which was more pronounced in the four benchmark structures. A linear trend between the ground truth and predicted value were frequently observed (which though does not affect the Pearson correlation metric). As a result, a shrinkage of variance of predicted score was also often observed together with the mean-shift (Supplementary Fig. 1d). As the Tiling Library data was standardized with mean 0 and unit standard deviation, we expect the predicted value in the testing folds should also approximately have 0 mean and unit standard deviation. The DCNN prediction accuracy in terms of Pearson correlation is close to IR+LSTM, while the average standard deviation of the predicted value is only about 0.75, in comparison to 0.91 in IR+LSTM (Supplementary Fig. 1c,d).

To remedy the mean shift and variance shrinkage issue, we applied a linear detrend layer in the end of all architectures. We first fit a linear regression between the ground truth and the predicted values in the training folds. The fitted regression function was applied to the predicted value by the given deep learning structure in the test folds for bias correction. This simple strategy is effective to remove the linear-trend and significantly improved the predicted score in MSE metric and mitigated the variance shrinkage issue particularly for the four benchmark structures (Supplementary Fig. 1c,d), though IR+LSTM maintains the superiority in both aspects. Model complexity-wise, the implemented IR+LSTM contains 94,485 Paras, and runs twice times more efficient than the DCNN which contains 178,161 Paras.

The finalized DNAcycP software tool is built on the best IR+LSTM model trained on the tiling library with detrending component.

**Supplementary Note 2: technical details for mouse data**

The track comparison between predicted and experimental cyclizability scores, i.e. C-score vs. loop-seq score are plotted through IGV genome browser (2). The loop-seq score track is extracted from ChrV Library of (3). The overall correlation between predicted and observed cyclizability is 0.773. For the extended region, please see tracks on IGV-Web app <https://tinyurl.com/ygd8co4t>.

The collection of well-annotated 3017 TSSs of S. cerevisiae genome was from (4). The nucleosome center positioning (NCP) scores, unique and redundant nucleosome maps were from (5). The genome coordinates were converted from Sac2 to Sac3 (Ensembl 104) using LiftOver app. The uniformly weighted occupancy is calculated by averaging NCP scores in +/- 73 bp region.

For S. cerevisiae genome, the +1 dyad is defined to be the center of nucleosome in the unique map within 150 bp downstream TSS, and -1 and +2 dyads as the nucleosome centers right before and after +1 dyad respectively. As the loop-score has a 7-bp resolution, it is linearly interpolated in creating Figure 2(b).

**Supplementary Note 3: technical details for loop-seq data set 5**

The FASTQ files of paired-end reads from L0 to L6 were downloaded from SRA/SRP047078 (6). For L0 and L6, PANDAseq (7) was used to assemble sequences, with arguments -p 20 -q 16 (trim forward primer and reverse primer), and -o 10 (minimum 10 bp overlap). Assembled sequences of exact 90 bp length were considered to be the valid ones. For L1-L5, the FASTQ files were first split based on corresponding barcodes (first 6 letters of each mate). Then sequences were assembled using PANDAseq with argument -p 26 -q 24.

For each sequence of length 90 bp, cyclizability scores were predicted for every 50 bp subsequence using a sliding window. For example, the C-score at position 25 or 65 represents the predicted intrinsic cyclizability scores of sequence fragment from position 1 to 50, or 41 to 90 respectively.

In plotting the AA/TT/AT/TA and GG/CC/GC/CG frequencies we found these dinucleotide motif frequencies have monotonic increasing trend along the sequence in L0 library. Following (6)**,** we corrected this motif frequency bias in L1-L6 carried from initial library selection (L0) by subtracting the average motif frequency in L0 at corresponding locations. The mean motif frequency along the sequence in L0 was then added back on top of the adjusted motif frequency at each position in each library.

**Supplementary Note 4: technical details for mouse data**

For mouse data, the NCP scores, unique maps and redundant maps were imported from (8). The genome coordinates were converted from version Mm8 to Mm39 (Ensembl 104) using LiftOver app. The center weighted occupancy is calculated through a smoothing window of Gaussian kernel with radius of 75 and standard deviation of 30.

For mouse genome, the “0” nucleosome dyad is defined as the center of the nucleosome in the unique map that covers the given TSS, i.e., nucleosome center resides within +/- 73 bp of TSS. The “-1” and “+1” nucleosome dyads refer to nucleosomes that do not cover a TSS, but their centers are within 221 bp upstream and downstream respectively.

**Reference**

1. Routhier, E., Pierre, E., Khodabandelou, G. and Mozziconacci, J. (2020) Genome-wide prediction of DNA mutation effect on nucleosome positions for yeast synthetic genomics. *Genome Res*, **31**, 317-326.

2. Thorvaldsdóttir, H., Robinson, J.T. and Mesirov, J.P. (2012) Integrative Genomics Viewer (IGV): high-performance genomics data visualization and exploration. *Briefings in Bioinformatics*, **14**, 178-192.

3. Basu, A., Bobrovnikov, D.G., Qureshi, Z., Kayikcioglu, T., Ngo, T.T.M., Ranjan, A., Eustermann, S., Cieza, B., Morgan, M.T., Hejna, M. *et al.* (2021) Measuring DNA mechanics on the genome scale. *Nature*, **589**, 462-467.

4. David, L., Huber, W., Granovskaia, M., Toedling, J., Palm, C.J., Bofkin, L., Jones, T., Davis, R.W. and Steinmetz, L.M. (2006) A high-resolution map of transcription in the yeast genome. *Proc Natl Acad Sci U S A*, **103**, 5320-5325.

5. Brogaard, K., Xi, L., Wang, J.-P. and Widom, J. (2012) A map of nucleosome positions in yeast at base-pair resolution. *Nature*, **486**, 496-501.

6. Rosanio, G., Widom, J. and Uhlenbeck, O.C. (2015) In vitro selection of DNAs with an increased propensity to form small circles. *Biopolymers*, **103**, 303-320.

7. Masella, A.P., Bartram, A.K., Truszkowski, J.M., Brown, D.G. and Neufeld, J.D. (2012) PANDAseq: paired-end assembler for illumina sequences. *BMC Bioinformatics*, **13**, 31.

8. Voong, L.N., Xi, L., Sebeson, A.C., Xiong, B., Wang, J.P. and Wang, X. (2016) Insights into Nucleosome Organization in Mouse Embryonic Stem Cells through Chemical Mapping. *Cell*, **167**, 1555-1570.e1515.

9. Chen, X., Xu, H., Yuan, P., Fang, F., Huss, M., Vega, V.B., Wong, E., Orlov, Y.L., Zhang, W., Jiang, J. *et al.* (2008) Integration of external signaling pathways with the core transcriptional network in embryonic stem cells. *Cell*, **133**, 1106-1117.

**Supplementary Figures**

**Figure S1.** **IR+LSTM model training and benchmark comparisons**. We carried out the model training in a 10-fold cross-validation framework: 9 folds used for training and 1 fold for test. The test fold is rotated among the 10 folds. Given a random assignment of 10 folds, 10 models are generated for a given architecture, each giving a prediction accuracy in terms of Pearson correction for the test fold. In addition, the 10 models were applied to the three external testing libraries (i.e., the other three libraries except the one used for training) to result in 10 prediction accuracy for each library. (a) Bar-plot of prediction accuracy of IR+LSTM model when trained under different libraries. Each column label indicates the library used in the training, and each row indicates the test data for prediction. The bar-plots in the diagonal position present the prediction accuracy averaged over the testing folds of the training data. The plots at off-diagonal positions present the average prediction accuracy for external libraries. The bandwidth of each bar presents the standard deviation of 10 predicted values. (b) Boxplots of prediction accuracy in Pearson correlation of different deep learning models trained based on tiling array. (c) Boxplot of MSE of prediction accuracy of different deep learning models trained based on tiling library. Each plots presents 10 MSE values evaluated based on the prediction on test folds of the training data, before and after detrending. (d) Same as in (c) but for the standard deviation of predicted score, before and after detrending.

**Figure S2.** **More transcription factor binding sites** (a) Mean C-score around thirteen selected transcription factor binding sites on mouse genome. The TF binding sites of mouse were from ChIP-Seq data (GSE11431, (9)). The exact TF binding sites were refined by scanning the ChIP-seq peak vicinity region using motif model of the TFs. (b) C-score (left panel) and NCP-score (right panel) averaged at the CTCF binding sites. Plotted are for quartiles based on CTCF binding site motif score. C-scores of Q2, Q3 are close to Q4, because their NCP scores are close.

**Supplementary Tables**

Table S1. Summary of C-sores on different species, from virus to mammal.

|  | Species | Min | 1^st^ quartile | Median | 3^rd^ quartile | Max | Mean | Std |
| --- | --- | --- | --- | --- | --- | --- | --- | --- |
| Virus | *phage T4* | -2.999 | -0.627 | -0.157 | 0.445 | 3.730 | -0.034 | 0.876 |
|  | *E. coli* | -2.859 | -0.558 | -0.173 | 0.364 | 3.983 | -0.023 | 0.782 |
| Archaea | *M. thermautotrophicus* | -2.933 | -0.289 | 0.083 | 0.592 | 3.951 | 0.215 | 0.753 |
|  | *T. kodakarensiss* | -3.005 | -0.427 | -0.032 | 0.534 | 3.928 | 0.138 | 0.817 |
| Yeast | *S. cerevisiae* | -3.155 | -0.587 | -0.118 | 0.497 | 3.981 | 0.009 | 0.892 |
|  | *S. pombe* | -3.250 | -0.652 | -0.171 | 0.448 | 3.949 | -0.051 | 0.911 |
| Mammal | *M. musculus* | -3.174 | -0.390 | 0.015 | 0.532 | 3.960 | 0.114 | 0.797 |
|  | *R. norvegicus* | -3.198 | -0.374 | 0.023 | 0.531 | 3.964 | 0.124 | 0.787 |
|  | *C. familiaris* | -3.185 | -0.424 | 0.008 | 0.532 | 3.978 | 0.092 | 0.837 |
|  | *M. mulatta* | -3.227 | -0.419 | 0.005 | 0.533 | 3.959 | 0.097 | 0.822 |
|  | *H. sapiens* | -3.194 | -0.420 | 0.006 | 0.536 | 4.018 | 0.099 | 0.826 |

**Table S1.** **Summary of C-sores on different species, from virus to mammal.** The C-score summaries of virus, archaea, and yeast are based on their whole genomes. For five mammal species, summaries are based on their chromosome 1 only.
